# Antisense RNA directed to the human papillomavirus type 16 E7 mRNA from herpes simplex virus type 1 derived vectors is expressed in CaSki cells and downregulates E7 mRNA

**DOI:** 10.1186/1743-422X-4-47

**Published:** 2007-06-04

**Authors:** Ilkka Kari, Stina Syrjänen, Bo Johansson, Piritta Peri, Bin He, Bernard Roizman, Veijo Hukkanen

**Affiliations:** 1 Department of Virology, Institute of Dentistry, University of Turku, Turku, Finland; 2 MediCity Research Laboratory, Institute of Dentistry, University of Turku, Turku, Finland; 3 Department of Oral Pathology, Institute of Dentistry, University of Turku, Turku, Finland; 4 The Marjorie B. Kovler Viral Oncology Laboratories, The University of Chicago, Chicago, IL, USA; 5 Department of Microbiology, University of Oulu, Oulu, Finland; 6 Department of Microbiology and Immunology, University of Illinois, Chicago, IL, USA; 7 Department of Clinical Virology, Karolinska University Hospital, Stockholm, Sweden

## Abstract

**Background:**

Human papillomavirus (HPV) infection is known to be the most important etiologic factor of cervical cancer. There is no HPV specific therapy available for treatment of invasive squamous cell carcinoma of the cervix and its precursor lesions. The present study elucidates the potential to use herpes simplex virus (HSV) derived vectors for expression of antisense RNA to HPV -16 E7 oncogene.

**Results:**

We have constructed replication competent, nonneuroinvasive HSV-1 vectors, deleted of the γ_1_34.5 gene. The vectors express RNA antisense to the first 100 nucleotides of the HPV-16 E7 gene. We assayed the ability of the antisense E7 vectors R5225 (*tk*-) and R5226 (*tk+*), to produce antisense RNA, as well as the consequent effects on E7 mRNA and protein levels in HPV-16 positive CaSki cells. Anti-E7 RNA was expressed by both constructs in a dose-dependent manner. Expression of HPV-16 E7 mRNA was downregulated effectively in CaSki cells infected with the *tk- *recombinant R5225 or with R5226. The *tk+ *recombinant R5226 was effective in downregulating E7 protein expression.

**Conclusion:**

We have shown that anti-E7 RNA expressed from an HSV vector could efficiently downregulate HPV-16 E7 mRNA and E7 protein expression in CaSki cells. We conclude that HSV vectors may become a useful tool for gene therapy of HPV infections.

## Background

Human papillomaviruses (HPVs) are small, non-enveloped DNA viruses that infect epithelial cells of skin and mucosa and replicate only in differentiating keratinocytes. Infection of the mucosa with high-risk HPV types is considered as the single most important etiological factor in cervical carcinogenesis. Particularly, HPV-16 is found in over 50% of squamous cell carcinomas of the uterine cervix [1,2]. Currently, there is no HPV specific therapy available for treatment of invasive squamous cell carcinoma of the cervix and its precursor lesions. However, there are promising results from prophylactic randomized HPV vaccination trials using virus-like particle vaccines against HPV -16 and -18 or HPV -6, -11, -16, and -18 [3].

E6 and E7 are the major oncogenic proteins produced by the cervical cancer associated HPVs [4,5]. Efficient keratinocyte immortalization requires cooperation of both proteins. Association of the E6 protein with p53 results in ubiquitin-dependent degradation of this tumor suppressor protein [6]. In addition to p53, E6 can bind to at least twelve other cellular proteins [7]. E7 can interact with the pRb tumor suppressor protein [8-11], which results in release of transcription factor E2F, leading to increased cell cycle progression. Continued expression of the E6 and E7 genes is necessary for the maintenance of the malignant phenotype [12]. Thus, the E6 and E7 gene products are important oncoproteins and feasible targets for anti-cancer therapies. CaSki cells, originally derived from a human cervical cancer [13], contain approximately 600 copies of HPV-16 DNA, and the E7 gene is continuously expressed in these cells.

Several approaches have been tested to inhibit E6 and E7 expression of HPV-16 and -18 in vitro. Antisense oligonucleotides have proven to be ineffective due to poor penetration and stability even with liposomes [14,15]. With hairpin antisense ribozymes an effective inhibition of HPV-16 E6/E7 immortalization has been reported [16]. Also retro- and adenoviral vectors producing antisense RNA have been used with potential approach to the therapy of HPV-16 positive cervical cancer [17,18].

Hybridization of antisense RNA with a complementary mRNA sequence leads to formation of untranslatable double-stranded RNA (dsRNA) molecules [19]. On the other hand, dsRNA is subject to degradation in eukaryotic cells [20]. Recently, the RNA interference (RNAi) technology has been tested against the HPV gene expression in cell lines [21,22]. RNAi against E6 and E7 has also been shown to enhance the chemotherapeutic effect of cisplatin in HPV-18 positive HeLa cells [23]. Successful inhibition of the E6 or E7 genes of HPV-16 or -18 has been achieved using transfection of siRNA [24-26] or short hairpin (sh) RNA expression plasmids [27] to HPV-positive cells. The siRNA injections have also been used for treatment of mouse tumor models [24,25]. As an alternative approach to inhibit the function of E6, Das and coworkers have demonstrated growth inhibition of HPV 16 E6-expressing cells by expressing p53 homologue p73beta, not subject to degradation by the E6 protein, from an adenoviral vector [28].

Genetically engineered herpes simplex viruses (HSVs) have been proposed to be used for treatment of human malignant tumors, such as malignant gliomas [29-32]. HSV has several advantages as a gene therapy vector. Its large genome contains up to 40 kbp of such genetic material which is nonessential in infections of certain cultured cells. Moreover, HSV has the ability to establish life-long latent infections, and express latency-associated RNA for tens of years [33,34]. The virulence and toxicity factors have been mapped in great detail [34]. The use of HSV vectors, deleted of the γ_1_34.5 gene, has proven safe in phase I studies in patients with gliomas [35,36]. It is conceivable that HSV vectors with deletion of the γ_1_34.5 gene would be advantageous in cancer virotherapy studies, since these viruses can not antagonize the effects of the PKR kinase, induced by double stranded RNA molecules in the infected cell [37].

To date, there has been no report on using replicative HSV vectors for gene therapy of papillomavirus infections. The present study focuses on the testing of a replication competent, nonneuroinvasive HSV-1 vector, lacking the γ_1_34.5 gene. The vector was designed to express RNA antisense to the first 100 nucleotides of the HPV-16 E7 gene, from the egr-1 promoter. We assayed the ability of the vector to produce antisense RNA and its effect on E7 mRNA and protein levels in cultures of CaSki cells, which carry the integrated DNA of HPV 16. The present study focused on the mRNA and protein changes in monolayer cultures, where the viruses cause lytic infection similarly to the wild type (wt) HSV-1.

## Results

### Construction and characterization of the recombinant viruses R5225 and R5226 containing the DNA sequence coding for RNA antisense to HPV-16 E7 ORF

The HSV viruses used in this study and their genotypes were: R5225 (γ_1_34.5-/*tk*-/antisense E7) and R5226 (γ_1_34.5-/*tk*+/antisense E7), and their control viruses R3617 (γ_1_34.5-/*tk*-), R3616 (γ_1_34.5-/*tk*+), and R3659 (γ_1_34.5-/Pα 27*tk*+) as described in the Methods section.

**Figure 1:**
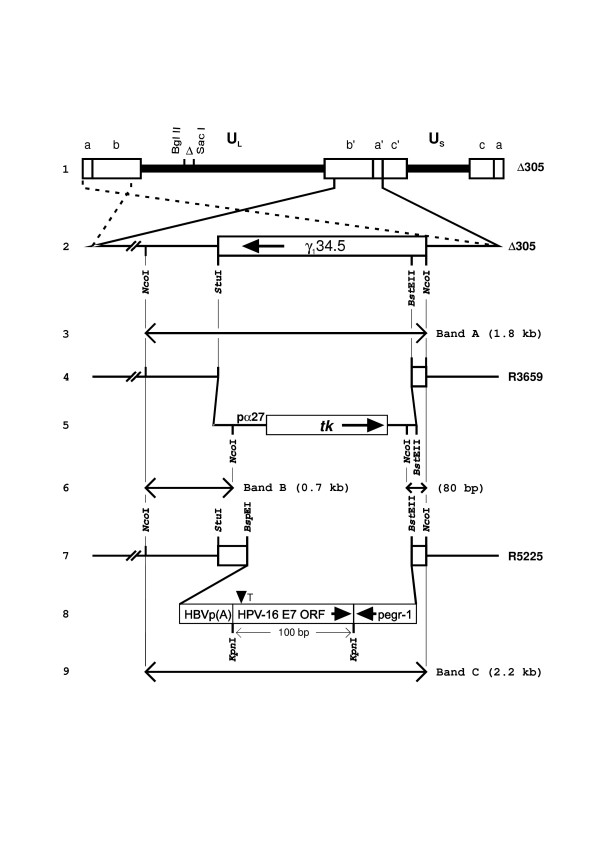
**Recombinant viruses**. Schematic representation of the sequence arrangements in the recombinant viruses used in this study. Line 1, the HSV-1(F)Δ 305 genome lacks the 501-bp *Bgl*II-*Sac*I sequence from the *tk *gene domain in the *Bam*HI Q fragment of HSV-1(F). Line 2, the domain of the γ_1_34.5 gene in the inverted repeat *b'a' *flanking U_L _sequence. The identical sequence in inverted orientation in the *ab *repeat is shown with dashed lines. Lines 4 and 5, sequence arrangement of the relevant domain of the R3659 recombinant. The *Stu*I-*Bst*EII fragment from the γ_1_34.5 domain was replaced with the chimeric α 27-*tk *gene. The substitution was made in both the *ab *and *a'b' *domains of the recombinant genome (not shown diagrammatically). Lines 7 and 8, sequence arrangement of the relevant domain of the R5225 recombinant. The α 27-*tk *gene of the R3659 recombinant was replaced with a cassette containing the first 100 bases of the HPV-16 E7 ORF in an antisense orientation under the egr-1 promoter, and the hepatitis B virus polyadenylation signal. An additional deoxythymidine nucleotide was inserted at the nucleotide 571 of the HPV-16 E7 ORF ("T") in order to introduce a frameshift and two stop codons in the sense orientation of the E7 ORF. The corresponding *tk*+ recombinant R5226 was constructed by placing the *tk *gene back to its natural location ("Δ ", line 1). The R3616 recombinant has a 1 kb deletion in both copies of the γ_1_34.5 gene, and the rest of the genome is intact. R3617 is similar to R3616 but is *tk *negative, having a 501 bp deletion in its *tk *gene similarly as in line 1. Lines 3, 6, and 9, expected sizes of bands generated by restriction enzyme digestion with *Nco*I and hybridized with digoxigenin-labeled 1.8 kb *Nco*I fragment of the *Bam*HI S; HSV-1(F), R3659, and R5225 would yield bands A, B, and C, respectively (see Fig. 2).

**Figure 2:**
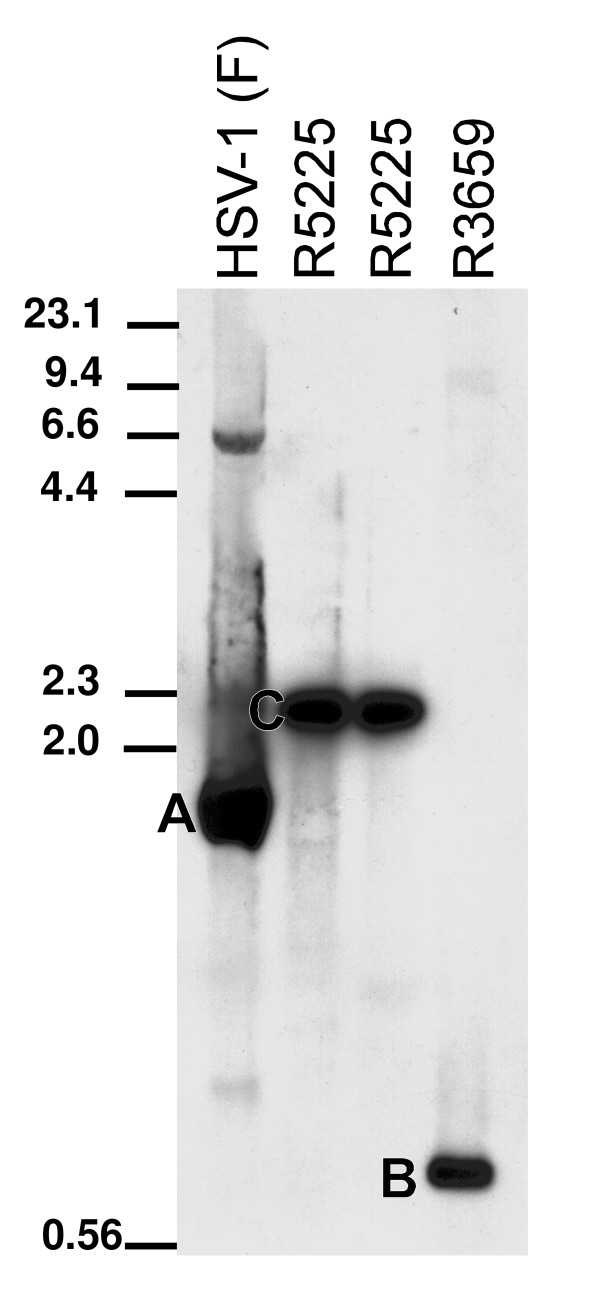
**Southern blot analysis of viral DNA**. Luminogram of electrophoretically separated, *Nco*I-digested and hybridized viral DNA fragments. Viral DNAs were prepared from large scale cultures of Vero cells infected with the recombinant viruses, digested with *Nco*I, electrophoretically separated on an agarose gel, transferred to a Zeta probe membrane, and hybridized with digoxigenin-labeled 1.8 kb *Nco*I fragment of the *Bam*HI S. The predicted sizes of the bands generated by the digestions shown in Fig. 1 are approximately 1.8 kb for band A, 0.7 kb for band B, and 2.2 kb for band C. The 80 bp band (Fig.1, line 6) was too small to be detected in this electrophoresis. Viral DNA from two different plaques of R5225 were analyzed (the two lanes labeled as R5225).

The procedures for construction of the recombinant viruses R5225 and R5226 are presented in Fig. 1 and described in the Methods section. Briefly, the first 100 nucleotides of the E7 gene of HPV-16 were cloned in antisense orientation in to a plasmid pRB 4878 [29] under the egr-1 promoter, flanked by sequences derived from the γ_1_34.5 gene of HSV-1 (F). An additional deoxythymidine nucleotide was inserted at the nucleotide position 571 of the HPV-16 E7 ORF in order to introduce a frameshift, introducing two stop codons in the sense orientation. The E7 antisense element-containing plasmid pRB5225 was cotransfected with DNA of the γ_1_34.5 deletion virus R3659, and the resultant recombinant virus was designated R5225. The luminescence images of the electrophoretically separated restriction fragments of viral DNA are shown in Fig. 2. The DNA fragments, detected by hybridization with digoxigenin-labeled 1.8 kb *Nco*I fragment of *Bam*HI S, corresponded with the patterns predicted in Fig. 1. The wt 1.8 kb *Nco*I subfragment of the *Bam*HI S represents the DNA sequence cloned in pRB4794. The 0.7 kb *Nco*I fragment present in the parental virus R3659 is designated as band B (Figs. 1 and 2) and is replaced by the 2.2 kb *Nco*I fragment C present in the recombinant virus R5225 (Figs. 1 and 2). The recombinant virus R5226 contains an identical insertion within the γ_1_34.5 domain, and in this virus the natural thymidine kinase (*tk*) gene has been repaired by cotransfection with a wt BamHI Q fragment containing plasmid. The Southern hybridization pattern of the *Bam*HI restriction fragments of R5226 viral DNA indicated the presence of the wt *Bam*HI Q fragment (data not shown). The transgenes in the viruses R5225 and R5226 were verified by nucleotide sequencing. Both R5225 and R5226 expressed anti-E7 RNA in Vero and CaSki cell cultures at 16 h.p.i., as detected by Northern blotting (not shown) and by quantitative RT-PCR (see below). In separate experiments we observed that the expression of the viral transgenes was highest at 16–20 h.p.i. in our cell culture setting, and the effects on the HPV-16 E7 mRNA were most evident at 16 h.p.i. (data not shown). Therefore the timepoint of 16 h.p.i. was selected for further studies. Both viruses caused a lytic infection and cytopathic effect in monolayer cultures of Vero and CaSki cells at late times post infection, beyond 24 h.p.i., when used at an m.o.i. of 1.0 or higher.

### R5225 and R5226 express anti-E7 RNA similarly in a dose-dependent manner, and downregulate HPV-16 E7 mRNA

**Figure 3:**
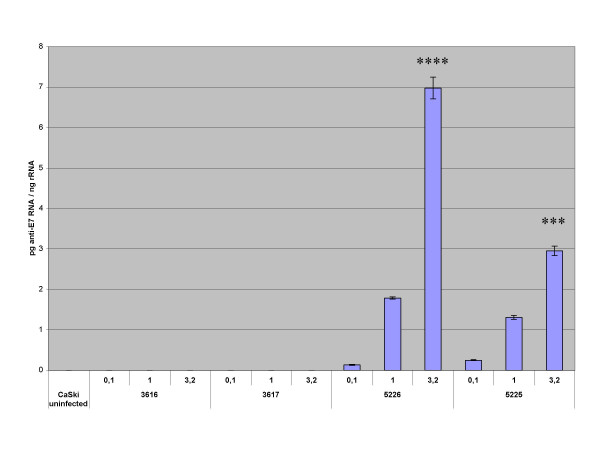
**Real-time RT-PCR analysis of anti-E7 RNA**. CaSki cells were infected with R3616, R3617, R5225, and R5226 at an m.o.i. of 0.1, 1.0, and 3.2, and harvested at 16 h.p.i. Total RNA was extracted and reverse transcription performed. Real-time PCR using the cDNA samples was performed as described in Materials and Methods. The diagram shows the amount of anti-E7 RNA, corrected for the 18S rRNA levels in these samples. Both R5225 and R5226 express antisense RNA in a dose-dependent manner. The statistical significances of the differences in anti-E7 copy numbers in comparison with uninfected CaSki cells are indicated by asterisks above the columns (***: p < 0.001; ****: p < 0.0001).

**Figure 4:**
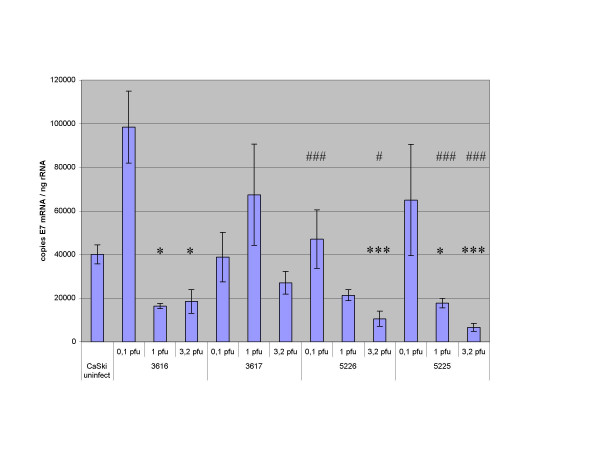
**Real-time RT-PCR analysis of HPV-16 E7 mRNA**. CaSki cells were infected with R3616, R3617, R5225, and R5226 at an m.o.i. of 0.1, 1.0, and 3.2, and harvested at 16 h.p.i. Total RNA was extracted and reverse transcription performed. Real-time PCR using the cDNA samples was done as described in Materials and Methods. The amount of E7 mRNA in HSV infected CaSki cells is given in copies of E7 mRNA molecules, corrected against rRNA levels in the same samples. The statistical significances of the differences in E7 copy numbers in comparison with uninfected CaSki cells are indicated by asterisks above the columns (*:p < 0.05; ***: p ≤ 0.001). The significances are shown for the cases with reduced E7 mRNA levels. The E7 mRNA values of the *tk+ *control R3616 were also compared with those of the *tk+ *antisense recombinant R5226 at the same m.o.i., and the *tk- *control R3617 values were compared with the *tk- *recombinant R5225 values at the same m.o.i.. The statistical significances in the pairwise comparisons for the reduced E7 mRNA levels are indicated by (#) above the respective columns (#: p < 0.05; ###: p ≤ 0.001).

For study of the antisense E7 RNA expression from the viruses R5225 and 5226, monolayer cultures of CaSki cells were infected with the recombinant viruses R3616, R5226, R3617, and R5225 at an m.o.i. of 0.1, 1.0, and 3.2, and harvested at 16 hours post infection. Real-time quantitative RT-PCR analyses were performed, using cellular RNA and the primers and probes listed in Table 1, to monitor antisense RNA production and resultant changes in E7 mRNA levels (Fig. 3 and 4). The experiment was performed in triplicate, with similar results. As control viruses, we used the *tk+*/γ_1_34.5- virus R3616 for the *tk+/*γ_1_34.5- antisense recombinant R5226, and the *tk-/*γ_1_34.5- virus R3617 as a control for the *tk-/*γ_1_34.5- recombinant R5225. We studied the changes in the E7 mRNA expression in comparison with the untreated CaSki cells, and made also pairwise comparisons of the effects of the corresponding control and antisense viruses. The outcome of these experiments was as follows:

**Table 1:** Oligonucleotides used for construction of the antisense plasmid and for real-time PCR

Purpose	Name	Sequence (5'-3')
pRB5225	E7ASp-FP	GCTTAGGGTACCATGCATGGA**T**GATACACCTA
construction	E7ASp-RP	GCTTAGGGTACCCTCTGAGCTGTCATTTAA
Real-time	HBVAS-FP	GAGAAGGGTCGTCCGCAGGAT
PCR	16E7AS-RP	CTCTGAGCTGTCATTTAATTGCTCATA
anti-E7	16E7AS-PRO	(6-FAM)-TGAATATATGTTAGATTTGCAACCAGAGACAACTGATCTCTACT-(TAMRA)
Real-time	16E7-FP	CAGCTCAGAGGAGGAGGATGAA
PCR	16E7-RP	CACACTTGCAACAAAAGGTTACAATATT
HPV-16 E7	16E7-PRO	(6-FAM)-CCAGCTGGACAAGCAGAACCGGAC-(TAMRA)

Abbreviations: AS, antisense; p, plasmid; FP, forward primer, RP, reverse primer; PRO, probe; FAM, 6-carboxyfluorescein; TAMRA, 6-carboxy-tetramethylrhodamine. The underlined base (T) in the primer "E7ASp-FP" is an insertion that introduces a frameshift and two stop codons in the sense orientation of the HPV-16 E7 ORF. The presence of the frameshift mutation in the pRB5225 plasmid was verified by sequencing. The primers and probes for the real-time RT-PCR except for "HBVAS-FP" were designed using Primer Express software (Applied Biosystems).

(i) Anti-E7 RNA was expressed by both the R5225 and R5226 constructs in a dose-dependent manner. R5226 was slightly more effective in producing antisense RNA. The control viruses R3616 and R3617 expressed no detectable anti-E7 RNA (Fig. 3). At m.o.i. of 3.2 the viruses R5225 and R5226 expressed significantly anti-E7 RNA (p < 0.001 and p < 0.0001, respectively, in comparison with untreated CaSki cells)

(ii) The *tk*- recombinant R5225 downregulated HPV-16 E7 mRNA expression effectively in CaSki cells (Fig. 4). CaSki cells infected with R5225 showed 74 and 75 per cent relative decrease in E7 mRNA levels compared to those infected with the control virus R3617 (*tk*-), at an m.o.i. of 1.0 and 3.2, respectively (p < 0.001; Fig. 4). In comparison with the untreated CaSki cells, R5225 decreased significantly the E7 mRNA levels at m.o.i. of 1 (p < 0.05) and 3.2 (p < 0.001).

(iii) The R5226 construct expressed more anti-E7 RNA than R5225 (Fig. 3). However, R5226 was slightly less effective in downregulating the HPV-16 E7 mRNA expression in CaSki cells, yet it was effective in reducing the E7 protein (below). R5226 was more effective than its control virus R3616 at an m.o.i. of 0.1 (p < 0.001) and 3.2 (p < 0.05), and the E7 mRNA was significantly reduced in comparison with untreated cells at m.o.i. of 3.2 (p < 0.001) (Fig. 4). The parental virus R3659 did not show significant effects on the E7 mRNA levels in CaSki cells even at the m.o.i. of 1.0 or 3.2 (a minor reduction to 84% and 76% of E7 mRNA amount of untreated CaSki cells, respectively; data not shown). The downregulation of E7 mRNA was not only a result of nonspecific decrease in all cellular mRNA levels, because we found that the cellular beta-actin mRNA levels were unaffected by the anti-E7 HSV recombinants even at high m.o.i. (data not shown). It is also noteworthy that the shown quantities of the E7 or anti-E7 molecules were presented as ratios to cellular rRNA.

(iv) HPV-16 E7 mRNA was downregulated also in CaSki cells infected with the control virus R3616 at higher m.o.i.s (p < 0.05), but not significantly with the *tk-*, γ_1_34.5 negative virus R3617 (Fig. 4). At low m.o.i. the R3616 virus rather induced the E7 mRNA expression. The reduction in E7 mRNA is most likely due to the general degradation of host cell mRNA pool, caused by the shutoff phenomenon in HSV infection *per se*.

### HPV-16 E7 protein expression was most efficiently downregulated with R5226

**Figure 5:**
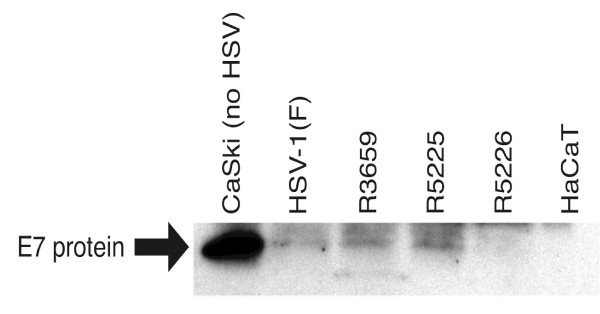
**Western blot of E7 protein**. Photograph of infected CaSki cell proteins electrophoretically separated in denaturing gels and reacted with a rabbit polyclonal antibody to HPV-16 E7 protein. HPV-16 positive CaSki cells were infected with R3659, R5225 or R5226 (1.0 p.f.u. per cell), harvested at 16 h.p.i., lysed, run on a 15% SDS-PAGE gel, transferred on a PVDF membrane, and probed first with the anti-HPV-16 E7 antibody and then with an HRP-conjugated anti-rabbit-Ig secondary antibody. The columns in the graph are: 1, CaSki cells with no HSV; 2 to 5, CaSki cells infected with wt HSV-1 (F), R3659, R5225, and R5226, respectively; 6, HaCaT cells (immortalized human keratinocytes with no HPV and thus no E7). The arrow indicates the location of the E7 protein in the gel.

For study of the E7 protein levels, CaSki cells were infected with wt HSV-1(F), R3659, R5225, and R5226 at an m.o.i. of 1.0 and 3.2 and harvested at the same time point as in the previous experiments. The immunoblotting assay was performed with a polyclonal anti-HPV-16 E7 antibody (Fig. 5) and a computerized image analysis was done to monitor E7 protein levels in infected CaSki cells (data not shown). Strong downregulation of E7 protein expression was observed in cells infected with the recombinant viruses. At an m.o.i. of 1.0, CaSki cells infected with R3659 showed a more than 10-fold decrease in E7 protein band intensity compared with uninfected CaSki cells (Fig. 5). Further, CaSki cells infected with the *tk*+ recombinant R5226 showed 81 per cent decrease in E7 band intensity compared with R3659. At 3.2 p.f.u. per cell, no E7 protein could be detected in cells infected with any of the viruses (data not shown).

## Discussion

Several approaches have been tested to inhibit the E6 and E7 expression of HPV-16 or -18. In our previous studies degradation of E7 mRNA could be achieved using antisense oligonucleotides directed to the E7 gene, though no effect on the E7 protein could be detected [15]. The penetration and stability of antisense oligonucleotides proved to be poor even with liposomal delivery [14]. Alvarez-Salas and coworkers have shown that anti-E6 oligodeoxynucleotides could inhibit growth of transplanted HPV-16 positive tumors in nude mice, but this required constant delivery of the oligonucleotides to the tumor tissue using osmotic pumps. They have also shown that cis-expression of an anti-E6 ribozyme and HPV-16 E6/E7 genes in normal human keratinocytes could efficiently prevent growth rate and immortalization [16,38]. Recently, siRNA [39] or sh RNA [27] have been used for successful silencing of HPV 16 E6 and E7 gene expression in CaSki and SiHa cell lines [22,25,27] or silencing of HPV-18 E6 and E7 in cervical cancer cells [24,26]. However, the siRNAs [24-26] or shRNA expression plasmids [27] were introduced into the cells by transfection, similarly to the ssDNA antisense oligonucleotides.

Recombinant adeno- and retroviruses are widely used as gene delivery systems in experimental tumor therapy both *in vitro *and *in vivo*. An adenoviral vector has been constructed expressing RNA antisense to HPV-16 E6 and E7 from the cytomegalovirus promoter [17]. The growth of SiHa cells infected with the recombinant adenovirus was significantly reduced. No data was given on the expression levels of E6 and E7 mRNAs. Interestingly, it was also found that the tumorigenicity of the infected SiHa cells totally disappeared in an *ex vivo *study in nude mice [17]. Choo and coworkers cloned full-length HPV-16 E7 cDNA in a retrovirus vector in reverse orientation to inhibit HPV-16 E7 in CaSki cells [18]. Similarly to our results, they found a reduction of HPV-16 E7 mRNA and protein expression after retroviral infection. Also a dose-dependent transduction of antisense HPV-16 E7 construct was able to inhibit and/or retard the tumorigenicity of the CaSki cells in nude mice [18].

The present study focused on the applicability of HSV vectors in gene therapy of oncogenic HPV-16 infection in cell culture. Effects on HPV oncogene mRNA expression were assessed with real-time RT-PCR, which is an excellent method for detection and quantitation of RNA species, in this case HPV-16 E7 mRNA and the anti-E7 RNA produced by the new recombinant HSV vectors. The real-time RT-PCR experiment showed that both recombinants expressed the desired antisense RNA in a dose-dependent manner, and particularly the *tk*- R5225 was constantly effective in downregulating HPV-16 E7 mRNA in CaSki cells. The relative reduction of E7 mRNA in cells infected with R5225 was considerable at an m.o.i. of 1.0 and 3.2. Infection with the *tk*+ recombinant R5226 downregulated E7 mRNA in CaSki cells at an m.o.i. of 3.2 (p < 0.001), and at an m.o.i. of 0.1 the downregulation was better than with the control virus R3616. At m.o.i. of 1.0 the experimental variation prevented reaching significance, but a clear E7 mRNA reduction is observed. The antisense sequence in our constructs, expressed from the egr-1 promoter, covered only the first 100 nucleotides of the E7 gene, however, our results show that an effective downregulation was achieved by using it. Our previous studies showed that the blockage of the 5'-end of the E7 sequence with antisense oligonucleotides was essential for degradation of HPV-16 E7 mRNA [15]. The egr-1 promoter has been suitable for transgene expression from similar HSV-1 vectors in many cell types [29] and it may be enhanced by irradiation. However, use of cell type- or cancer-specific promoters would still improve our approach.

The powerful shutoff of protein expression in the host cell caused by the herpes simplex virus infections influenced the interpretation of the specific effect of the antisense RNA, produced by the recombinant viruses, on the E7 expression in monolayer cultures of CaSki cells. The results of our quantitative RT-PCR were, however, standardized to the levels of cellular RNA. We studied also the expression of cellular beta-actin mRNA and found that it was unchanged in infections with the recombinant anti-E7 HSV at moi's of 1.0 and 3.2, suggesting that the observed effects on the E7mRNA were not result of a generalized decrease in all cellular RNAs. The HSV-1 (F) wild type virus infection was not used as control for the E7 mRNA determinations, due to the presence of the intact γ_1_34.5 gene. Rather, we used the corresponding γ_1_34.5 negative viruses R3616 (*tk+)*, and R3617 (*tk-) *for comparisons. We studied also the effects of the parental virus R3659 (Pα 27*tk+) *on the E7 expression, though its genomic structure is different from our other *tk+ *viruses. The changes in E7 mRNA levels are quickly reflected in E7 protein expression, since the half-life of the HPV-16 E7 protein produced by recombinant baculoviruses in Sf-21 insect cells at +37°C is less than 30 minutes [40]. In Western blotting studies of CaSki cells, at an m.o.i. of 1.0, the amount of E7 protein in CaSki cells infected with the *tk*+ recombinant R5226 was grossly reduced and was about five times less than with R3659 (Fig. 5). The effects of the HSV vectors on cellular proliferation or apoptosis could not be properly studied in our model, because the differences in these phenomena accumulate at much later timepoints of experiments, such as on days 5–9 post treatments [25]. Also, our HSV vectors harbor the majority of the anti-apoptotic genes of wt HSV [34], which limits the study of the effects of the transgenes on apoptosis. Because the transgene sequences are identical, the slight differences between the effects of R5225 and R5226 on the E7 mRNA levels may be partly due to the presence of the intact U_L_23 and U_L_24 genes in the correct genomic location in R5226, or to some possible yet unknown secondary mutations. The *tk*+ recombinant R5226 had no clear growth advantage in the CaSki cells in monolayer cultures.

The need for viral vectors as carriers of the antisense elements to tissues and cells in vivo remains in spite of the novel oligonucleotide strategies. The potential to infect non-dividing cells and to establish long-term transgene expression, using suitable promoters (e.g. pLAT), give herpes simplex viruses advantages as gene therapy vectors. More research will be needed to determine the qualities of an optimal herpes simplex vector for RNA-mediated therapy of HPV infection. Viral vectors have already been used for delivery of short RNA molecules to certain cell types [41]. Herpes simplex virus can express microRNA from its genome [42] and it is likely that HSV can be used as a vector for expression of microRNA to cells infected with HPV. Our next studies will concentrate on determining the effects of the vectors on cell growth and differentiation, as well as on constructing new, optimized HSV vectors. The γ_1_34.5 gene deletion mutants of HSV may exhibit less lytic and less virulent infection pattern in vivo and in highly organized tissue culture settings, which are an object of a further study. As such, our vectors will be subject to further attenuation, now that the effect of the transgene has been established. The tropism of HSV to the genital epithelium, which is the site of HPV infection, renders the attenuated vectors derived from HSV-1 as potential vehicles for the RNA expression elements.

## Conclusion

As of now, there are no previous reports on the applicability of HSV vectors in treatment of HPV infections. The present study introduces two new recombinant herpes simplex virus vectors that express RNA antisense to HPV-16 E7. We showed that anti-E7 RNA expressed from an HSV vector could efficiently downregulate HPV-16 E7 mRNA expression. Although the vector needs further optimization, we conclude that the results of the present study suggest that HSV vectors may become a powerful tool for gene therapy of HPV infections.

## Methods

### Cells and viruses

CaSki cells were obtained from the American Type Culture Collection (ATCC, Manassas, VA, USA). The cells were propagated in Dulbecco's modified Eagle's medium (DMEM) supplemented with 1% non-essential amino acids, 50 μg/ml streptomycin, 100 U/ml penicillin, and 10% fetal bovine serum (FBS).

HSV-1 (F) is the prototype HSV-1 strain used in this study. The recombinant virus R3659, described previously [43], lacks the *Bgl*II *-Sac*I subfragment of the *Bam*HI Q fragment, encoding the *tk *and U_L_24 genes. In R3659 the *Bst*EII-*Stu*I subfragment of the *Bam*HI S fragment, encoding the γ_1_34.5 and ORF P genes, is replaced by a chimeric gene consisting of the coding domain of the *tk *gene fused to the α 27 promoter (see Fig. 1).

The virus R3616 has a 1 kb deletion in both copies of the γ_1_34.5 gene [43,44], and the rest of the genome is intact. R3617 is similar to R3616 but is thymidine kinase negative, having a 501 bp deletion in its *tk *gene similarly as in Fig. 1, line 1.

### Plasmids

The plasmid pRB4878 has been described elsewhere [29]. Briefly, it contains the egr-1 promoter, polylinker site and the hepatitis B virus polyadenylation signal cloned within the *Bsp*EI-*Bst*EII deletion of the γ_1_34.5 gene (Fig. 1).

Plasmid pRB5225, containing the first 100 bases of the HPV-16 E7 gene in antisense orientation under the egr-1 promoter, was made by insertion of a 100-bp PCR product, digested with *Kpn*I, into the *Kpn*I site in the polylinker sequence of the pRB4878 plasmid (Fig. 1). The PCR reaction was performed using HPV-16 DNA-containing CaSki cellular DNA as template and *Pfu*I DNA polymerase (New England Biolabs, Beverly, MA, USA). The cloning PCR primers E7ASp-FP and E7ASp-RP are shown in Table 1. An additional deoxythymidine nucleotide was inserted in the E7ASp-FP primer in order to introduce a frameshift at the nucleotide position 571 of the HPV-16 E7 ORF, introducing two stop codons in the sense orientation of the HPV-16 E7 ORF. The correct sequence of the insertion in the pRB5225 plasmid and in the viruses was verified by sequencing (Table 1).

### Construction of recombinant viruses

The antisense E7-RNA-expressing virus R5225 was constructed by cotransfection of rabbit skin cells with the pRB5225 plasmid and R3659 viral DNA, using protocols described previously [29]. The selection for the resulting recombinant *tk*- viruses was performed on 143TK- cells overlaid with DMEM containing 5% fetal bovine serum and 100 μg/ml of bromodeoxyuridine (BUdR). The selection was done by three successive rounds of plaque purification under BUdR, followed by preparation of viral DNA and stocks from Vero cell cultures infected with selected viral plaques, as described elsewhere [45]. The corresponding *tk*+ virus R5226 was constructed according to methods described previously [29]. The purified DNA of the virus R5225 was cotransfected into rabbit skin cells with the plasmid pRB165 containing the entire *Bam*HI-Q fragment. The selection for *tk*+ viruses was done by successive rounds of HAT selection in 143TK- cells overlaid with HAT medium (DMEM supplemented with 5% FBS, hypoxanthine, aminopterin, and thymidine), followed by plaque purifications. The viral stocks and DNA were prepared from Vero cell cultures infected with plaque purified viruses.

### Analysis of viral DNA

The large scale preparation of viral DNA was done from roller cultures of Vero cells, infected with selected plaque stocks of the recombinant viruses. The DNA was purified using NaI gradients [46]. The correct structure of the insert was verified by hybridization of the electrophoresed, Southern blotted *Nco*I-digested viral DNA fragments with digoxigenin-labeled 1.8 kb *Nco*I fragment of the *Bam*HI S fragment (derived from the plasmid pRB4794; see [43]) (Fig. 2) or with digoxigenin-labeled oligonucleotide E7ASp-FP used for cloning the HPV-16 E7 insert (data not shown). The presence of the DNA insert in the recombinant genome was also verified by PCR of the isolated viral DNA preparates using the insert-specific primers E7ASp-FP and E7ASp-RP. The DNA preparates of R5226, digested with *Bam*HI, were analyzed for the presence of the intact *Bam*HI Q fragment by Southern hybridization with the digoxigenin-labeled pRB165 plasmid, containing the *Bam*HI Q fragment of HSV-1 (F) DNA.

### Infection of CaSki cells and production of cDNA

Trypsinized CaSki cells were plated on 6-well tissue culture plates (Falcon, Becton Dickinson Labware, Franklin Lakes, NJ, USA) and let reach 80% confluency. The cells were infected with either R3617, R3659, R5225, R3616, or R5226 using either 0.1, 1.0, or 3.2 plaque forming units (p.f.u.) per cell. After adsorption (1 h, 37°C), monolayers were washed with PBS and overlaid with DMEM containing 10% FBS. CaSki cells were harvested at 16 h post infection and total RNA was extracted using the TRIZOL Reagent and treated with DNase I for 15 minutes in room temperature as described by the manufacturer (Life Technologies, Paisley, UK). Reverse transcription was performed with 1 μg of total RNA using the "1^st ^Strand cDNA Synthesis Kit" (Amersham Pharmacia Biotech Inc., Uppsala, Sweden) and random hexamer primers for 1 h at 37°C. In time series experiments the level of transgene expression was found to be highest at 16–20 h.p.i., and the effects on the E7 mRNA in CaSki cells were most evident at 16 h.p.i. Therefore the 16 h.p.i was selected as a timepoint for further experiments. The HSV vectors caused cytopathogenicity in the cell monolayer cultures at late timepoints, beyond 24 hpi.

### Real-time PCR

Real-time PCR using the cDNA samples was performed with the "ABI Prism 7700 Sequence Detection System" and the "TaqMan Universal PCR Master Mix" (Applied Biosystems, Foster City, CA, USA). The amplification conditions were: initial incubations for 2 min at 50°C and for 10 min at 95°C, followed by PCR cycling using a two step cycle at 95°C for 15 sec and 60°C for 60 sec for a total of 40 cycles. The primers and probes are shown in Table 1. The primers and probes for this experiment except for HBVAS-FP were designed using the "Primer Express" software (Applied Biosystems). Specific detection of anti-E7 cDNA was achieved with an upstream primer (HBVAS-FP) specific for the hepatitis B virus polyadenylation sequence present only in the antisense RNA. A standard curve for anti-E7 cDNA was obtained by amplification of a dilution series of cDNA produced from RNA transcribed *in vitro *from a pGEM-T plasmid containing the insert of pRB5225. A standard curve for E7 cDNA was obtained by amplification of a dilution series of cDNA from CaSki cell total RNA. All samples were also analyzed with the 18 S rRNA Kit (PE Biosystems, Warrington, UK), and the anti-E7 and E7 values were corrected using the values from these amplifications. At least three "no template control" reactions were included in each run. As a control for cellular mRNA changes during the HSV vector infections we studied the cellular beta-actin mRNA by quantitative RT-PCR as described previously [47]. The statistical analyses were performed with SAS software using the Dunnett generalized linear model procedure.

### Protein electrophoresis and immunoblotting

CaSki cells were grown as described above in 20 cm^2 ^cell culture dishes (Nalge Nunc International, Roskilde, Denmark) to 80% confluency. A similar culture of HPV negative HaCaT cells (immortalized human keratinocytes) was grown to yield a control sample not containing E7 protein. CaSki cells were infected with R3659, R5225 and R5226 at a multiplicity of infection (m.o.i.) of 1.0 and 3.2 and harvested at 16 hours post infection. The cells were lysed by incubation in 50 μl of buffer containing 150 mM NaCl, 50 mM Tris (pH 8.0), 5 mM EDTA, 1% Nonidet-P 40, 2 mM dithiothreitol, and 2 mM PMSF for 30 minutes on ice. The lysate was centrifuged at 12000 × *g *for 30 minutes at +4°C. The supernatant with proteins was removed and stored at -70°C.

The samples, containing 30 μg of protein, were suspended in a 2 × sample buffer (100 mM Tris-HCl (pH 6.8), 200 mM dithiothreitol, 4% SDS, 0,2% bromophenol blue, 20% glycerol), boiled for 4 minutes and run on a 15% SDS-PAGE. The proteins were transferred electrophoretically to "Hybond-P" PVDF membrane (Amersham Life Science, Buckinghamshire, UK) in a running buffer containing 25 mM Tris, 192 mM glycine, and 20% v/v methanol. Equal loading was verified with Ponceau S staining. The membranes were blocked with a buffer containing 1 × PBS, 0,1% Tween-20, and 5% non-fat dried milk for 1 h and then probed with a rabbit polyclonal anti-HPV-16 E7 antibody (a gift from Dr. Massimo Tommasino). The membranes were washed with PBS containing 0.1% Tween-20 before and after incubation with the primary antibody, 2 times for 10 min and 5 times for 5 min, respectively. The membranes were then incubated with an HRP-conjugated anti-rabbit-Ig secondary antibody (Dako, Glostrup, Denmark) for 1 h and detected using ECL+Plus Western blotting detection reagents (Amersham Pharmacia Biotech, Uppsala, Sweden). Computerized image analysis was performed to quantitate the intensities of the signals with "Microcomputer Imaging Device version M4" (Imaging Research, St. Catharines, Ontario, Canada).

## Competing interests

The author(s) declare that they have no competing interests.

## Authors' contributions

IK participated in the design of the study, carried out the experimental infections, the RT-PCR and immunoblotting assays and drafted the manuscript. SS participated in the design and coordination of the study and helped to draft the manuscript. BJ designed the RT-PCR assays and analyzed their results. BR and BH have contributed to the design and construction of the recombinant viruses. PP participated in the experimental infections and cell cultures and in the statistical analyses. VH conceived of the study, participated in its design and coordination, designed and constructed the antisense HSV vectors, and helped to draft the manuscript. All authors have read and approved the final manuscript.
